# Supplementary material for: Tamoxifen and Fertility in Women with Breast Cancer: A Systematic Review on Reproductive Outcomes and Oncological Safety of Treatment Interruption
Source: Int J Mol Sci. 2025 Apr 17;26(8):3787. doi: 10.3390/ijms26083787 (PMC12028241; doi:10.3390/ijms26083787)
Supplement: Supplementary file 1 [file ijms-26-03787-s001.zip › ijms-3578133-supplementary.pdf]

Supplementary Table S1. Risk of bias for the selected studies.
The green and yellow dots represent a low and moderate risk of bias, respectively.

Study (First Author, Year)	Risk of Bias Tool	Confounding-Randomization Bias	Performance Bias - Bias in Classification of Exposures	Detection - Measurement Bias	Attrition Bias - Missing data	Reporting Bias
**Partridge et al., 2023 (POSITIVE)**	RoB 2	🟢	🟢	🟢	🟢	🟢
**Shandley et al., 2017**	NOS	🟡	🟡	🟡	🟡	🟡
**Nye et al., 2017**	NOS	🟢	🟡	🟢	🟢	🟢

NOS, Newcastle-Ottawa Scale; RoB 2: Risk of Bias 2 tool
